# Pharmacological HIF-inhibition attenuates postoperative adhesion formation

**DOI:** 10.1038/s41598-017-13638-z

**Published:** 2017-10-13

**Authors:** Moritz J. Strowitzki, Alina S. Ritter, Praveen Radhakrishnan, Jonathan M. Harnoss, Vanessa M. Opitz, Marvin Biller, Julian Wehrmann, Ulrich Keppler, Jana Scheer, Markus Wallwiener, Thomas Schmidt, Alexis Ulrich, Martin Schneider

**Affiliations:** 1 Department of General, Visceral and Transplantation Surgery, University of Heidelberg, Heidelberg, Germany; 2 Department of General Gynaecology and Obstetrics, University of Heidelberg, Heidelberg, Germany; 3 Present Address: Cancer Immunology, Genentech, Inc., South San Francisco, USA

## Abstract

Peritoneal adhesions represent a common complication of abdominal surgery, and tissue hypoxia is a main determinant in adhesion formation. Reliable therapeutic options to reduce peritoneal adhesions are scarce. We investigated whether the formation of postsurgical adhesions can be affected by pharmacological interference with hypoxia-inducible factors (HIFs). Mice were treated with a small molecule HIF-inhibitor, YC-1 (3-[5′-Hydroxymethyl-2′-furyl]-1-benzyl-indazole), or vehicle three days before and seven days after induction of peritoneal adhesions or, alternatively, once during induction of peritoneal adhesions. Pretreatment or single intraperitoneal lavage with YC-1 significantly reduced postoperative adhesion formation without prompting systemic adverse effects. Expression analyses of cytokines in peritoneal tissue and fluid and *in vitro* assays applying macrophages and peritoneal fibroblasts indicated that this effect was cooperatively mediated by various putatively HIF-1α-dependent mechanisms, comprising attenuated pro-inflammatory activation of macrophages, impaired recruitment and activation of peritoneal fibroblasts, mitigated epithelial-mesenchymal-transition (EMT), as well as enhanced fibrinolysis and impaired angiogenesis. Thus, this study identifies prevention of postsurgical peritoneal adhesions as a novel and promising field for the application of HIF inhibitors in clinical practice.

## Introduction

Peritoneal adhesions represent a common long-term complication of abdominal surgery, and can lead to bowel obstruction, female infertility or chronic pain^1^. Despite the apparent clinical and health-economic relevance, no reliable strategies for the prevention of adhesion formation could yet be established in general surgery^1^.

Development of permanent adhesions is a multistep process, comprising a macrophage-driven inflammatory response, formation of a fibrinous exudate, recruitment of fibroblasts which become activated to form myofibroblasts, excess collagen fibre deposition and subsequent vascularization^2–5^. These processes occur in a low oxygen environment (hypoxia), which critically modulates inflammation and healing^3,6,7^. On the molecular level, responses to hypoxia are orchestrated by hypoxia-inducible factors (HIFs), consisting of an oxygen-dependent α - (HIF-1α, HIF-2α) and an oxygen-independent β-subunit^8^. HIF-α-subunits are constitutively expressed and rapidly degraded in normoxia^8^. In hypoxia, however, HIF-1α and HIF-2α are stabilized and form active transcription complexes. Those bind to hypoxia response elements (HRE) in the promoter region of numerous downstream target genes, which collectively mount the adaptive response to hypoxia.

Due to their implications in various diseases involving tissue hypoxia, the HIFs represent attractive drug targets^8,9^. Various HIF-inducers and -inhibitors have been developed and tested in preclinical conditions^9^. YC-1 (3-(5′-Hydroxymethyl-2′-furyl)-1-benzyl-indazole) is a prototype small molecule inhibitor of HIF, which down-regulates HIF-1α- and HIF-2α protein levels, and inhibits the formation of transcriptionally active HIF complexes^10^.

HIFs are not only important modulators of inflammation, fibrosis and epithelial-mesenchymal-transition (EMT), but likewise implicated in peritoneal adhesion formation^6,11–13^. We therefore hypothesized that therapeutic interference with HIF signalling attenuates postoperative adhesion formation. Here, we assessed whether therapeutic HIF-inhibition using YC-1 reduces peritoneal adhesions.

## Results

### Intraperitoneal YC-1-treatment reduces peritoneal adhesion formation

**Figure 1:**
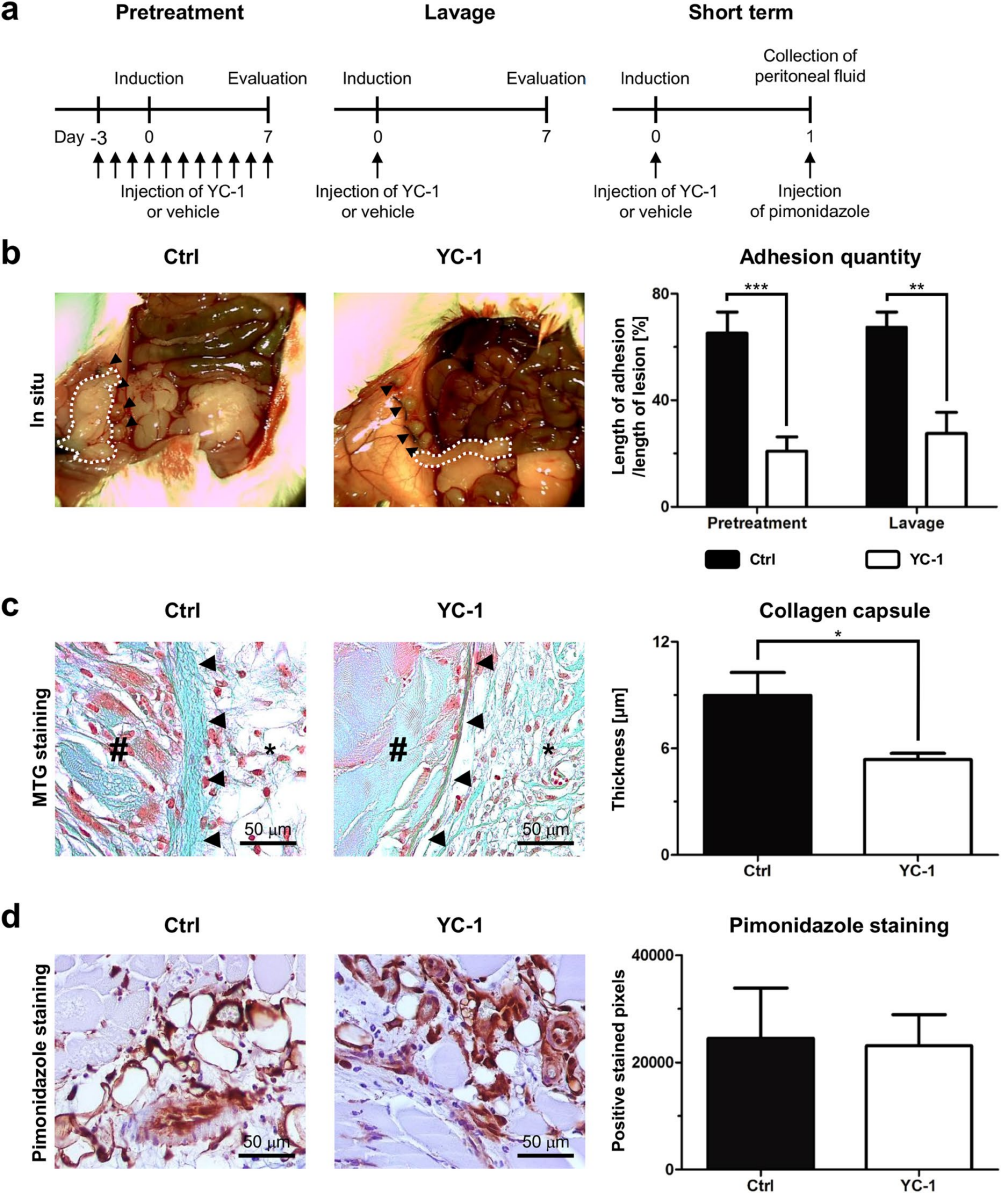
YC-1 reduces peritoneal adhesion formation. (**a**) Experimental schedules. Pretreatment with YC-1 was performed for three days before surgical adhesion induction, and continued for seven days until evaluation (*left*). Alternatively, single intraoperative lavage with YC-1 was performed upon adhesion induction (*middle*). To assess short-term effects, pimonidazole injection and subsequent sampling of tissue along with peritoneal fluid were performed 24 h after adhesion induction and intraoperative lavage with YC-1 (*right*). (**b**) *Left:* Representative images of ischemic buttons (arrowheads) and postoperative adhesions (dotted white lines) on postoperative day (POD) 7 following pretreatment with YC-1 or vehicle (Ctrl). *Right:* Quantification of postoperative adhesions on POD7 after pretreatment or single intraoperative lavage with YC-1 or vehicle (Ctrl) (***P ≤ 0.001, **P ≤ 0.01, *n* = 9). (**c**) *Left:* Representative Masson-Trichrom-Goldner stainings, revealing the thickness of collagen capsules (arrowheads) in between the necrotic core (#) and adjacent peritoneum (asterisks) of ischemic buttons in mice pretreated with YC-1 or vehicle (Ctrl). *Right:* Histomorphometric quantification, revealing reduced maximal capsule thickness in YC-1-pretreated mice (*P ≤ 0.05, *n* = 7). Scale bars = 50 μm. (**d**) Representative pimonidazole immunostainings (*left*) and histomorphometric quantification (*right*), revealing comparable extents of hypoxic tissue areas within ischemic buttons of vehicle- (Ctrl) and YC-1-treated animals 24 h after adhesion induction (*P* > 0.05, *n* = 4). Scale bars = 50 μm.

In a first approach to assess the effects of YC-1 on postoperative adhesion formation, treatment with YC-1 (20 mg/kg BW i.p.) was initiated three days prior to adhesion induction applying the ischemic buttons model, and continued until evaluation on postoperative day seven (“pretreatment” scheme, Fig. 1a, *left*). In a second experimental setting, YC-1 was administered to the open abdomen as a single intraoperative dosage (“lavage” scheme, Fig. 1a, *middle*). In both settings, application of YC-1 strikingly reduced postoperative adhesion formation (Fig. 1b). Further assessment using an established score confirmed that pretreatment or lavage with YC-1 significantly altered the quality of adhesions towards a less stable phenotype (Table 1)^11^. Masson-Trichrome-Goldner-staining confirmed that the fibrous capsule surrounding the necrotic core of the ischemic buttons was significantly thinner upon YC-1-pretreatment, indicating anti-fibrotic effects (Fig. 1c). Importantly, postoperative pimonidazole-immunolabelling (Fig. 1a, *right*) revealed that intracellular hypoxia occurred to similar extent in ischemic buttons of YC-1- and control-treated mice (Fig. 1d), indicating that YC-1’s anti-fibrotic effects were truly due to an altered peritoneal response.

**Table 1:** Macroscopic grading of postoperative adhesions in mice treated with YC-1 or vehicle (DMSO) alone.

Score	Pretreatment				P-value	Lavage				P-value
	Ctrl		YC-1			Ctrl		YC-1		
	n	(%)	n	(%)		n	(%)	n	(%)	
**Extent**					0.007*					0.160*
0 – no adhesions	0	(0.0)	1	(12.5)		0	(0.0)	1	(14.3)	
1 – 1 – 25%	0	(0.0)	5	(62.5)		1	(12.5)	3	(42.9)	
2 – 26 – 50%	3	(33.3)	2	(25.0)		2	(25.0)	3	(42.9)	
3 – 51 – 75%	3	(33.3)	0	(0.0)		3	(37.5)	0	(0.0)	
4 – 76 – 100%	3	(33.3)	0	(0.0)		2	(25.0)	0	(0.0)	
**Type**					0.008*					0.650*
0 – no adhesions	0	(0.0)	1	(12.5)		0	(0.0)	1	(14.3)	
1 – filmy	0	(0.0)	1	(12.5)		1	(12.5)	2	(28.6)	
2 – dense	1	(11.1)	5	(62.5)		1	(12.5)	1	(14.3)	
3 – capillaries present	8	(88.9)	1	(12.5)		6	(75.0)	3	(42.9)	
**Tenacity**					0.267*					0.140*
0 – no adhesions	0	(0.0)	1	(12.5)		0	(0.0)	1	(14.3)	
1 – easily fall apart	0	(0.0)	1	(12.5)		0	(0.0)	2	(28.6)	
2 – require traction	1	(11.1)	2	(25.0)		2	(25.0)	2	(28.6)	
3 – require sharp dissection	8	(88.9)	4	(50.0)		6	(75.0)	2	(28.6)	
**Total**	**Mean**	**SEM**	**Mean**	**SEM**	**0.004** ^**#**^	**Mean**	**SEM**	**Mean**	**SEM**	**0.014** ^**#**^
	8.78	±0.32	5.00	±0.87		8.13	±0.52	4.86	±1.08	

*Ctrl* control group; *SEM* standard error of the mean; *statistical analysis by Fisher’s exact test; ^*#*^statistical analysis by Student’s t-test.

Remarkably, YC-1-treatment caused neither significant weight loss (Supplementary Fig. 1a and b), nor deficient wound healing. Indeed, pretreatment with YC-1 neither affected the wound breaking strength of laparotomy scar tissue, nor did it alter the amount of collagen deposits within laparotomy scars (Supplementary Fig. 1c–e). Assessment of serum transaminases and creatinine-levels revealed that i.p. lavage with YC-1 (at a dosage that significantly reduced adhesion formation, Supplementary Fig. 1f) did not induce hepato- or nephrotoxicity (Supplementary Table S1). In addition, YC-1-treatment did not affect postoperative plasma levels of EPO, a HIF-target (Supplementary Table S1). Consistently, neither erythrocyte counts, nor haemoglobin or haematocrit values were altered upon YC-1-treatment (Supplementary Table S1). The expression of HIF target genes in organs such as the liver or the heart was likewise unaltered after i.p. lavage with YC-1 (Supplementary Fig. 1g and h).

Thus, pretreatment or single intraperitoneal lavage with YC-1 strikingly reduced postoperative adhesion formation, without prompting overt adverse effects.

### YC-1-treatment ameliorates pro-adhesive inflammatory responses

**Figure 2:**
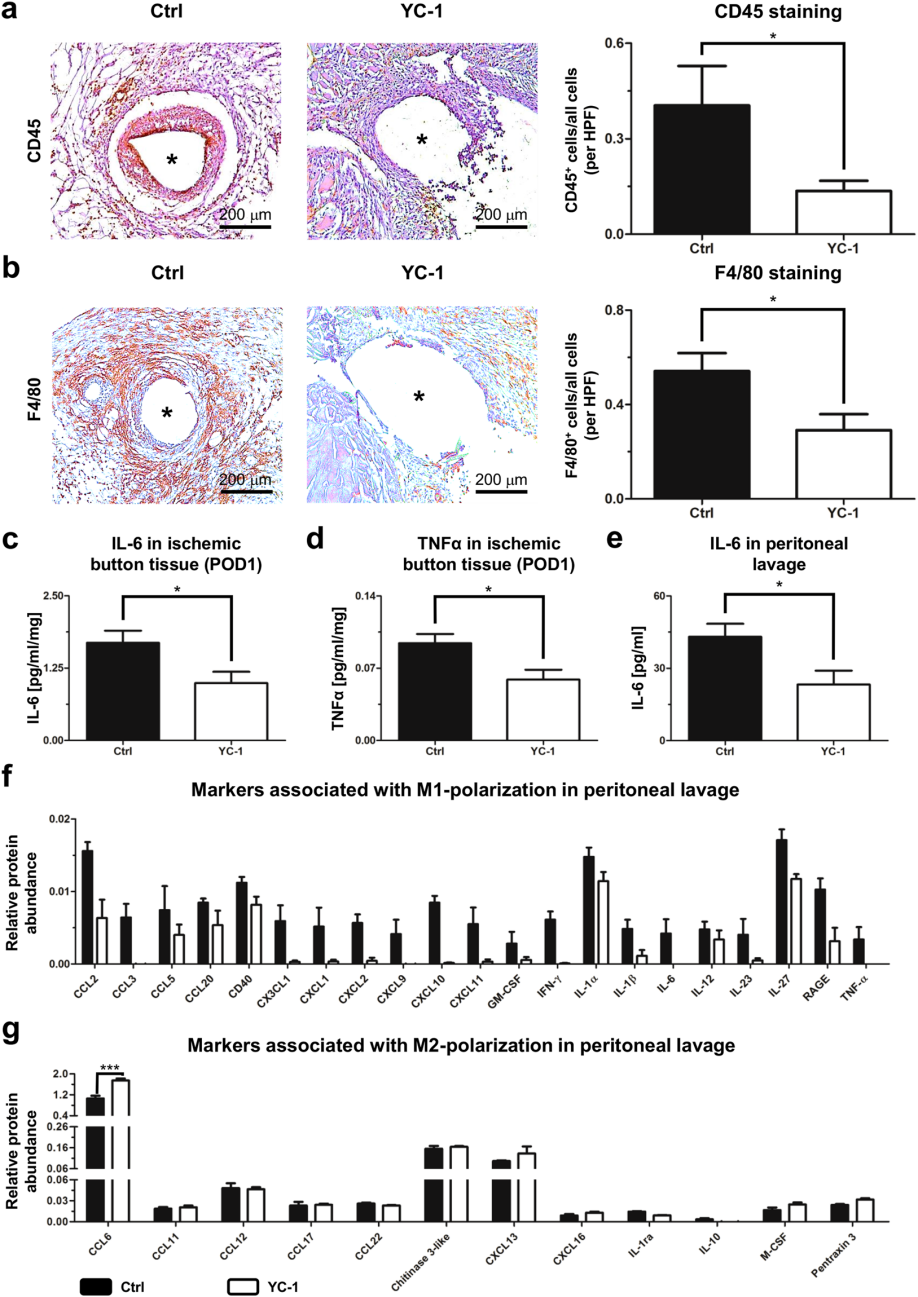
YC-1 ameliorates peritoneal inflammation in vivo. (**a**) Representative CD45 immunostainings (*left*) and histomorphometric quantification (*right*) of leukocytes within ischemic buttons of mice pretreated with YC-1 or vehicle (Ctrl), seven days after adhesion induction. (**b**) Representative F4/80 immunostainings (*left*) and histomorphometric quantification (*right*) of macrophages within ischemic buttons of mice pretreated with YC-1 or vehicle (Ctrl). Asterisks in (**a**,**b**) indicate positions of sutures (extracted) within ischemic buttons (see also Supplementary Fig. 5; *HPF* high power field; *P ≤ 0.05, *n* = 7 in (**a**,**b**). Scale bars = 200 μm. (**c**,**d**) ELISA of IL-6 (**c**) and TNFα (**d**) in tissue lysates of ischemic buttons, harvested 24 h after adhesion induction from Ctrl- and YC-1-treated animals (*P ≤ 0.05, *n* = 5) (**e**) ELISA-based quantification of IL-6 levels in peritoneal lavage fluid, harvested 24 h after adhesion induction (*P ≤ 0.05, n = 6). (**f**,**g**) Antibody array, revealing the relative abundance of M1- (**f**) and M2-macrophage-associated cytokines (**g**) in peritoneal fluid of mice pretreated with YC-1 or vehicle (Ctrl), one day after adhesion induction (***P ≤ 0.001, *n* = 4; ANOVA with Bonferroni post-hoc tests).

Immunolabelling of inflammatory cells was performed in order to assess whether reduced adhesion formation in YC-1-treated animals was accompanied by an attenuated inflammatory response. Indeed, seven days after adhesion induction the accumulation of CD45-positive leukocytes (Fig. 2a) and F4/80-positive macrophages (Fig. 2b) within peritoneal scar tissue was significantly attenuated after YC-1-pretreatment.

The role of macrophages in peritoneal adhesion formation is two-faced: pro-inflammatory (M1-polarized) macrophages support, while immunomodulatory (M2-polarized) macrophages attenuate it^4^. To test whether YC-1-treatment ameliorates adhesion formation via attenuated M1-polarization of peritoneal macrophages, we analysed the abundance of M1-specific cytokines in peritoneal scar tissue and in peritoneal fluid, isolated one day after adhesion induction (Fig. 1a, *right*). ELISA-based protein expression analysis revealed that protein concentrations of the M1 macrophage-secreted, pro-inflammatory cytokines IL-6 and TNFα were significantly reduced within peritoneal scar tissue of YC-1-treated mice (Fig. 2c and d). Consistently, IL-6 protein levels were significantly reduced in peritoneal fluid from YC-1-treated animals (Fig. 2e). Further analysis applying an antibody array likewise indicated generally reduced abundance of M1 macrophage-secreted, pro-inflammatory cytokines in peritoneal fluid of YC-1-treated mice (Fig. 2f; albeit this effect lost significance upon Bonferroni correction for multiple comparisons). By contrast, the abundance of M2 macrophage-secreted cytokines appeared to be either unaltered or increased in peritoneal fluid from YC-1-treated mice (Fig. 2g), collectively indicating a shift from M1- to M2-macrophage polarization.

**Figure 3:**
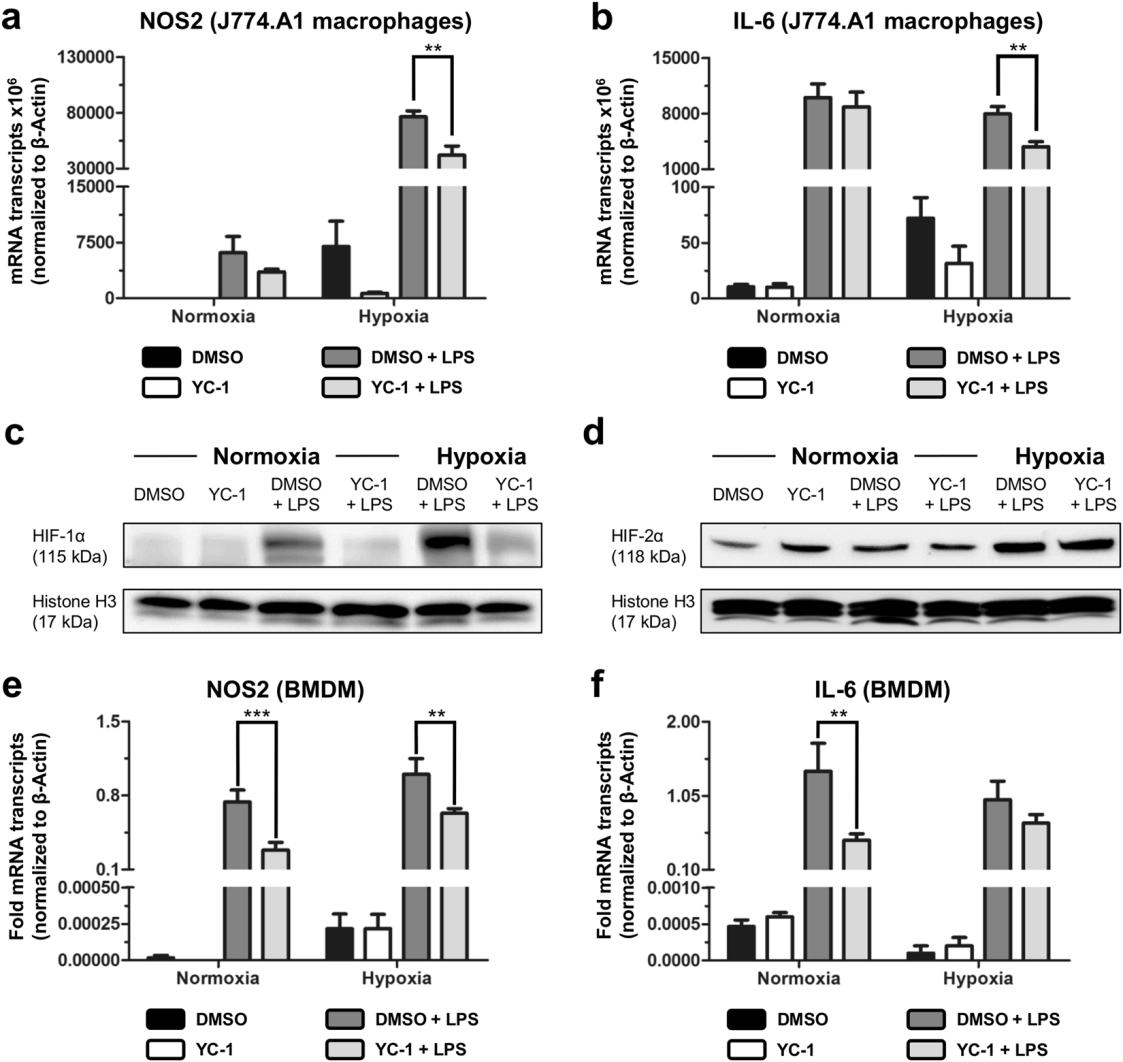
YC-1 attenuates pro-inflammatory differentiation of macrophages. (**a**,**b**) qRT-PCR analyses, revealing mRNA expression of the pro-inflammatory M1-differentiation markers NOS2 (**a**) and IL-6 (**b**) in murine J774A.1 macrophages under normoxia (left bars) and hypoxia (right bars). LPS exposure was carried out to induce M1-polarization in presence of YC-1 or vehicle control (DMSO; **P ≤ 0.01, *n* = 9). (**c**,**d**) Western blots of nuclear extracts, revealing protein levels of HIF-1α (**c**) and HIF-2α (**d**) in J774.A1 macrophages treated with vehicle (DMSO) or YC-1 under normoxic (*left*) or hypoxic (*right*) culture conditions. Simultaneous LPS-treatment was performed to induce pro-inflammatory activation. Note LPS-induced stabilization of HIF-1α- (**c**), but not HIF-2α-levels (**d**), and reversal of LPS-induced HIF-1α-stabilization by YC-1 (**c**). Histone H3 was used as a loading control. (**e**,**f**) qRT-PCR analyses, revealing mRNA expression of the pro-inflammatory M1-differentiation markers NOS2 (**e**) and IL-6 (**f**) in BMDMs under normoxia (left bars) and hypoxia (right bars). LPS exposure was carried out to induce M1-polarization in presence of YC-1 or vehicle control (DMSO; ***P ≤ 0.001, **P ≤ 0.01, *n* = 6).

To verify that YC-1-treatment attenuates pro-inflammatory M1-polarization of macrophages, murine J774.A1-monocytes were differentiated into macrophages^14^, and subsequently treated with LPS to induce M1-polarization in presence of YC-1 or vehicle control (DMSO) *in vitro*. Under normoxic conditions, LPS-stimulation expectedly caused a marked up-regulation of mRNA transcripts encoding the M1-markers iNOS (*NOS2*) and IL-6, which was non-significantly reduced by additional YC-1-treatment (Fig. 3a and b, *left bars*). Under hypoxia, LPS-triggered induction of these M1-markers was more pronounced than in normoxia, and significantly attenuated upon YC-1-treatment (Fig. 3a and b, *right bars*). We likewise assessed whether YC-1-treatment affects phagocytic activity, a key feature of M1-macrophages. Immunofluorescence labelling and quantification of phagocytosed bacterial particles revealed that the phagocytic activity of J774.A1 macrophages was slightly reduced upon YC-1-treatment at hypoxic culture conditions (Supplementary Fig. 2a and b). Western blotting of nuclear extracts indicated that these effects of YC-1 in J774.A1 macrophages were mediated by its property to antagonize HIF-1α. Indeed, LPS-induced up-regulation of nuclear HIF-1α protein in these cells was reverted by simultaneous YC-1-treatment in both normoxia and hypoxia (Fig. 3c). By contrast, nuclear HIF-2α protein levels remained mostly unaffected upon treatment with LPS and YC-1 (Fig. 3d).

Comparable findings were obtained when analysing the effects of YC-1 on primary bone marrow-derived macrophages (BMDMs; Supplementary Fig. 2c)^14^: YC-1-treatment attenuated LPS-induced expression of the M1-polarization markers iNOS (*NOS2*) and IL-6 (Fig. 3e and f) in BMDMs, and slightly decreased the phagocytic activity of these cells (Supplementary Fig. 2d and e).

Collectively, these findings indicate that YC-1-treatment alleviates postoperative adhesion formation by attenuating HIF-1α-induced M1-polarization of macrophages.

### YC-1-treatment attenuates EMT and activation of fibroblasts

Adhesion formation depends on epithelial-mesenchymal-transition (EMT) of mesothelial cells (MC), and on stimulation of peritoneal fibroblasts to form myofibroblasts^15,16^. Both processes are augmented by M1-polarized macrophages^17,18^.

**Figure 4:**
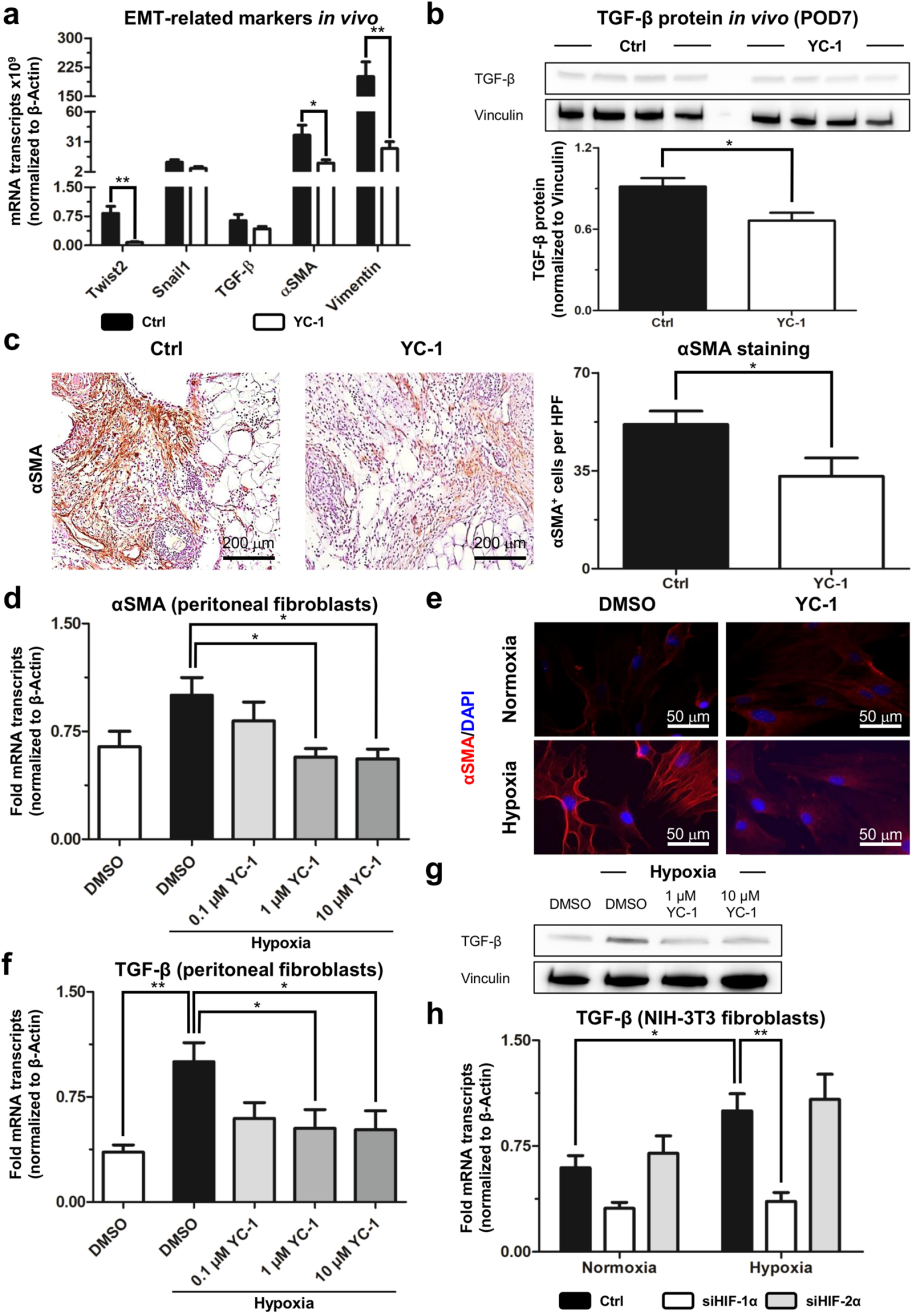
YC-1 attenuates EMT and fibroblast activation. (**a**) qRT-PCR analysis, revealing mRNA expression levels of EMT-associated genes (Twist2, Snail1, TGF-β) and myofibroblast markers (αSMA, Vimentin) in peritoneal adhesion tissue from mice pretreated with YC-1 or vehicle (Ctrl), seven days after adhesion induction (**P ≤ 0.01, *P ≤ 0.05, *n* = 8). (**b**) Western blot (*top*) and densiometric quantification (*bottom*), revealing TGF-β protein abundance in tissue lysates of ischemic buttons derived from YC-1- or Ctrl-treated mice, Vinculin was used as loading control (*P ≤ 0.05, *n* = 4). (**c**) Representative αSMA immunostainings (*left*) and histomorphometric quantification (*right*) of myofibroblasts within ischemic buttons of mice pretreated with YC-1 or vehicle (Ctrl), seven days after adhesion induction (*HPF* high power field; *P ≤ 0.05, *n* = 6). Scale bars = 200 μm. (**d**) qRT-PCR, revealing mRNA expression of the myofibroblast marker, αSMA, in peritoneal fibroblasts treated with vehicle (DMSO), or different dosages of YC-1 in hypoxic culture conditions. Note dose-dependent reduction of hypoxia-induced αSMA expression upon YC-1 treatment (*P ≤ 0.05, *n* = 6). (**e**) Representative immunocytochemistry for αSMA protein (red) and DAPI (cell nuclei, blue) in primary peritoneal fibroblasts treated with vehicle (DMSO) or YC-1 at normoxic (*left panels*) or hypoxic (*right panels*) culture conditions. Note reduction of hypoxia-induced αSMA protein expression upon YC-1 treatment. Scale bars = 50 μm. (**f**) qRT-PCR, revealing suppression of hypoxia-induced TGF-β mRNA expression in primary peritoneal fibroblasts upon treatment with YC-1 (**P ≤ 0.01, *P ≤ 0.05, *n* = 6). (**g**) Immunoblot revealing TGF-β protein levels in primary peritoneal fibroblasts treated with vehicle (DMSO) or YC-1 under normoxic (*left*) or hypoxic (*right*) culture conditions. Note hypoxic up-regulation of TGF-β, and reversal by YC-1. Vinculin (*bottom*) served as loading control. (**h**) qRT-PCR, revealing that expression of TGF-β mRNA in NIH-3T3 fibroblasts is suppressed by siRNA-mediated interference with HIF-1α (siHIF-1α), but not HIF-2α (siHIF-2α) under normoxic (*left bars*) and hypoxic (*right bars*) culture conditions (*P ≤ 0.05, **P ≤ 0.01, *n* = 6).

To assess putative effects of YC-1-treatment on adhesion-related EMT *in vivo*, we analysed the expression of various EMT-mediating factors in ischemic peritoneal button tissue, harvested seven days after adhesion induction. qRT-PCR revealed that transcript expression of twist-related protein 2 (Twist2, a HIF-1α target^19^) was significantly reduced by 10-fold in mice pre-treated with YC-1 compared to control-treated animals (Fig. 4a). mRNA expressions of Snail1 (an EMT-inducing HIF-1α target^20^) and TGF-β (a major stimulus for both EMT and fibroblast activation^21^) were likewise (albeit non-significantly) attenuated upon YC-1-treatment (Fig. 4a). Moreover, TGF-β protein expression was significantly reduced in ischemic buttons from YC-1-treated mice (Fig. 4b). mRNA expression levels of the mesenchymal marker vimentin, and the myofibroblast marker αSMA were likewise significantly decreased in adhesion tissue of YC-1-treated mice (Fig. 4a). Consistently, immunohistochemistry revealed a significantly reduced abundance of αSMA-positive activated fibroblasts within ischemic buttons of YC-1-treated mice (Fig. 4c).

We likewise analysed the abundance of proteins fostering adhesion-related fibroblast activation and EMT in peritoneal lavage fluid, harvested 24 hours after YC-1-treatment and adhesion induction. Concentrations of epidermal growth factor (EGF), hepatocyte growth factor (HGF), and, most strikingly, fibroblast growth factor 1 (FGF-1) were reduced in YC-1-treated compared to control-treated animals (Supplementary Fig. 3a). Conversely, the abundance of IGF-binding proteins (IGFBPs), which putatively exert anti-adhesion effects by inhibiting pro-adhesive properties of insulin-like growth factor (IGF)^22^, was enhanced upon YC-1-treatment (Supplementary Fig. 3b). Collectively, these data indicate that YC-1-treatment modulates the expression of various mediators of EMT and fibroblast activation towards a less fibrogenic phenotype.

To further elucidate the effects of YC-1 on fibroblast activation, we established primary cultures of murine peritoneal fibroblasts, and confirmed their fibroblast-phenotype by immunostaining for fibroblast-specific protein 1 (FSP-1; Supplementary Fig. 4a). When exposed to hypoxia, these cells expectedly up-regulated mRNA-expression of αSMA, indicating differentiation into pro-fibrogenic myofibroblasts (Fig. 4d). Strikingly, YC-1 dose-dependently abolished this effect. Indeed, treatment with 1 or 10 μM YC-1 reverted hypoxic overexpression of αSMA transcripts (Fig. 4d) and αSMA protein (Fig. 4e) to levels observed in normoxic fibroblasts. Hypoxic up-regulation of TGF-β, a potent autocrine stimulator of fibroblast activation, was similarly reverted upon YC-1-treatment of peritoneal fibroblasts on the mRNA (Fig. 4f) as well as on the protein level (Figure 4g)^23^. In an attempt to determine whether this effect was attributable to YC-1’s inhibitory effects towards HIF, we assessed nuclear HIF-1α- and HIF-2α protein levels in peritoneal fibroblasts. Indeed, YC-1 decreased hypoxia-induced protein abundance of HIF-1α (Supplementary Fig. 4b), whereas HIF-2α levels remained unaffected by YC-1 treatment (Supplementary Fig. 4c). Comparable results were observed when assessing the effects of YC-1-treatment on the abundance of nuclear HIF-1α and HIF-2α in embryonic fibroblasts (NIH-3T3 cell line; Supplementary Fig. 4d and e). We further measured the expression of TGF-β transcripts in embryonic fibroblasts (NIH-3T3 cell line), which were exposed to hypoxia upon simultaneous, siRNA-mediated silencing of either HIF-1α or HIF-2α (Supplementary Note 1; Supplementary Fig. 4f and g). Consistent with our findings in primary peritoneal fibroblasts, TGF-β expression of NIH-3T3 fibroblasts was significantly induced upon hypoxia (Fig. 4h). Knockdown of HIF-1α, but not of HIF-2α, completely abolished hypoxic up-regulation of TGF-β-expression in these cells (Fig. 4h), thus recapitulating the effects of YC-1-treatment.

Collectively, these findings suggest that YC-1-treatment reduces peritoneal adhesion formation by interfering with EMT and fibroblast activation, likely by antagonizing HIF-1α-mediated effects.

### Effects of YC-1-treatment on peritoneal fibrinolysis and angiogenesis

**Figure 5:**
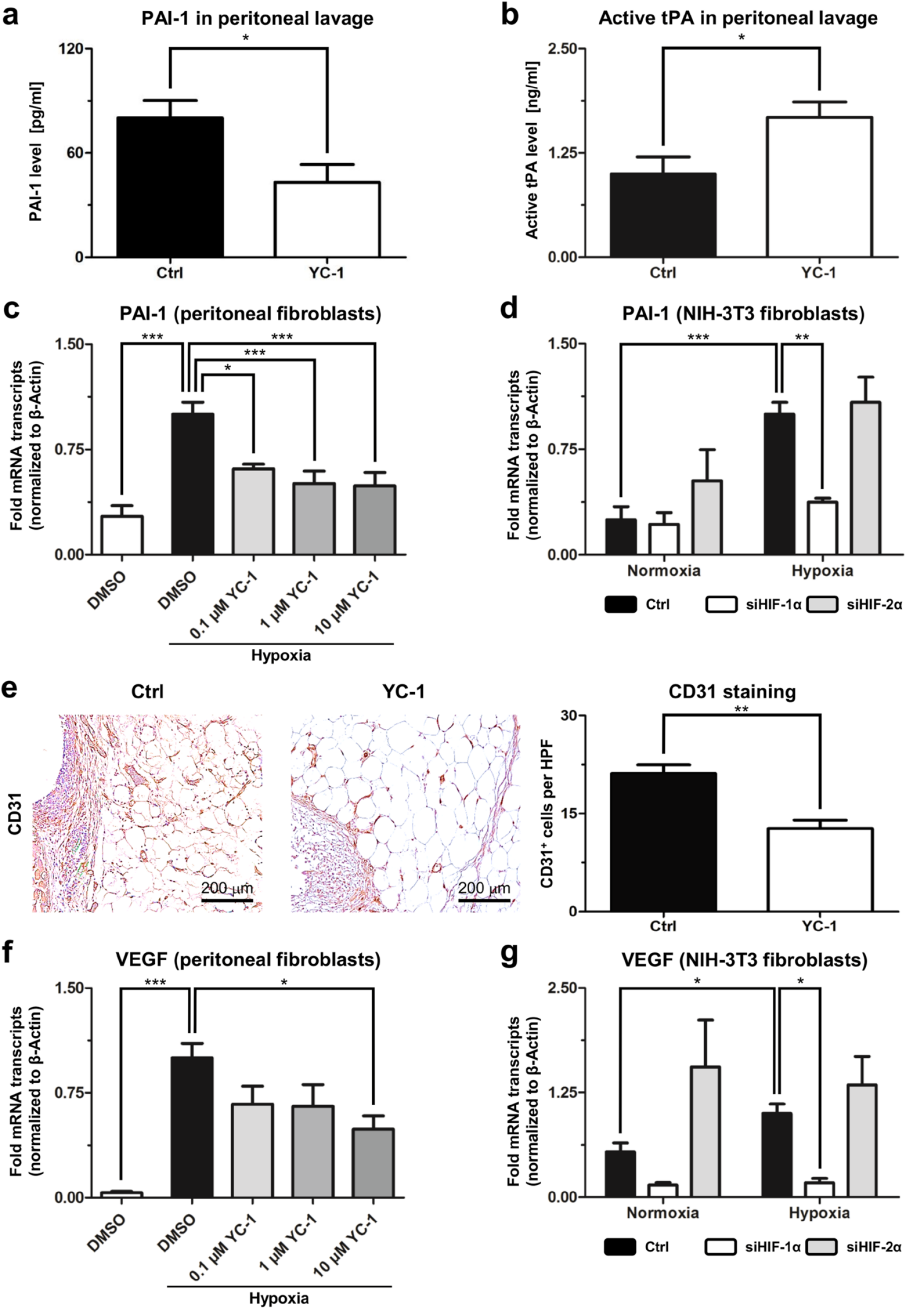
YC-1 enhances fibrinolysis and attenuates angiogenesis. (**a**,**b**) ELISA measurements, revealing concentrations of PAI-1 protein (**a**) and active tPA (**b**) in peritoneal fluid of mice treated with YC-1 or vehicle (Ctrl), one day after adhesion induction (*P ≤ 0.05, *n* = 6). (**c**) qRT-PCR, revealing suppression of hypoxia-induced PAI-1 mRNA levels in primary peritoneal fibroblasts upon treatment with YC-1 (*P ≤ 0.05, **P ≤ 0.01, *n* = 6). (**d**) qRT-PCR, revealing that hypoxia-induced expression of PAI-1 mRNA in NIH-3T3 fibroblasts is suppressed by siRNA-mediated interference with HIF-1α (siHIF-1α), but not HIF-2α (siHIF-2α) (***P ≤ 0.001, **P ≤ 0.01, n = 6; ANOVA). (**e**) Representative CD31 immunostainings (*left*) and histomorphometric quantification (*right*) of blood vessels within ischemic buttons of mice pretreated with YC-1 or vehicle (Ctrl), seven days after adhesion induction (*HPF* high power field; **P ≤ 0.01, *n* = 6). Scale bars = 200 μm. (**f**) qRT-PCR, revealing suppression of hypoxia-induced VEGF mRNA levels in primary peritoneal fibroblasts upon treatment with YC-1 (***P ≤ 0.001, *P ≤ 0.05, *n* = 6; ANOVA). (**g**) qRT-PCR, revealing that hypoxia-induced expression of VEGF mRNA in NIH-3T3 fibroblasts is suppressed by siHIF-1α, but not siHIF-2α (*P ≤ 0.05, n = 6).

We next sought to assess whether YC-1-treatment likewise affects fibrinolysis. An imbalance between fibrinogenesis and fibrinolysis represents a key feature underlying postoperative adhesion formation^3,24^. Fibrinolytic degradation of early adhesions relies on the activity of tissue plasminogen activator (tPA), which is inhibited by plasminogen activator inhibitor (PAI-1, a HIF-1α target^25^). Assessment one day after adhesion-induction revealed that PAI-1 protein was significantly less abundant in peritoneal fluid from YC-1-treated mice than in that from control animals (Fig. 5a). Consistently, levels of active tPA were significantly increased in peritoneal fluid from YC-1-treated mice (Fig. 5b), collectively indicating increased fibrinolytic potential.

We likewise determined the expression of PAI-1 in primary peritoneal fibroblasts. Consistent with the findings outlined above, hypoxic up-regulation of PAI-1 mRNA in peritoneal fibroblasts could be significantly attenuated upon YC-1-treatment *in vitro* (Fig. 5c). Hypoxic peritoneal fibroblasts also secreted significantly less PAI-1 protein following YC-1 treatment (Supplementary Fig. 4i). Hypoxic upregulation of PAI-1 expression could expectedly be confirmed in NIH-3T3 fibroblasts (Fig. 5d), and could be reverted by simultaneous, siRNA-mediated knockdown of HIF-1α, but not HIF-2α (Fig. 5d; Supplementary Note 1; Supplementary Fig. 4f and g). Collectively, these findings suggest that YC-1 inhibits HIF-1α to attenuate PAI-1-secretion by fibroblasts.

Finally, we addressed putative effects of YC-1-treatment on angiogenesis, a HIF-dependent mechanism necessary for stable adhesion formation^26^. CD31-immunostaining revealed that the density of newly formed blood vessels was significantly reduced in peritoneal scar tissue from mice pre-treated with YC-1 compared to animals undergoing control-treatment (Fig. 5e). *In vitro*, YC-1-treatment consistently attenuated hypoxia-driven upregulation of vascular endothelial growth factor (VEGF, a HIF-target^9^) in primary peritoneal fibroblasts (Fig. 5f). Similar to PAI-1 and TGF-β, hypoxic up-regulation of VEGF transcripts in NIH-3T3 fibroblasts (see above and Supplementary Note 1) could be significantly reverted by interference with HIF-1α but not HIF-2α, suggesting a link to HIF-1α-inhibition via YC-1 (Fig. 5g).

## Discussion

**Figure 6:**
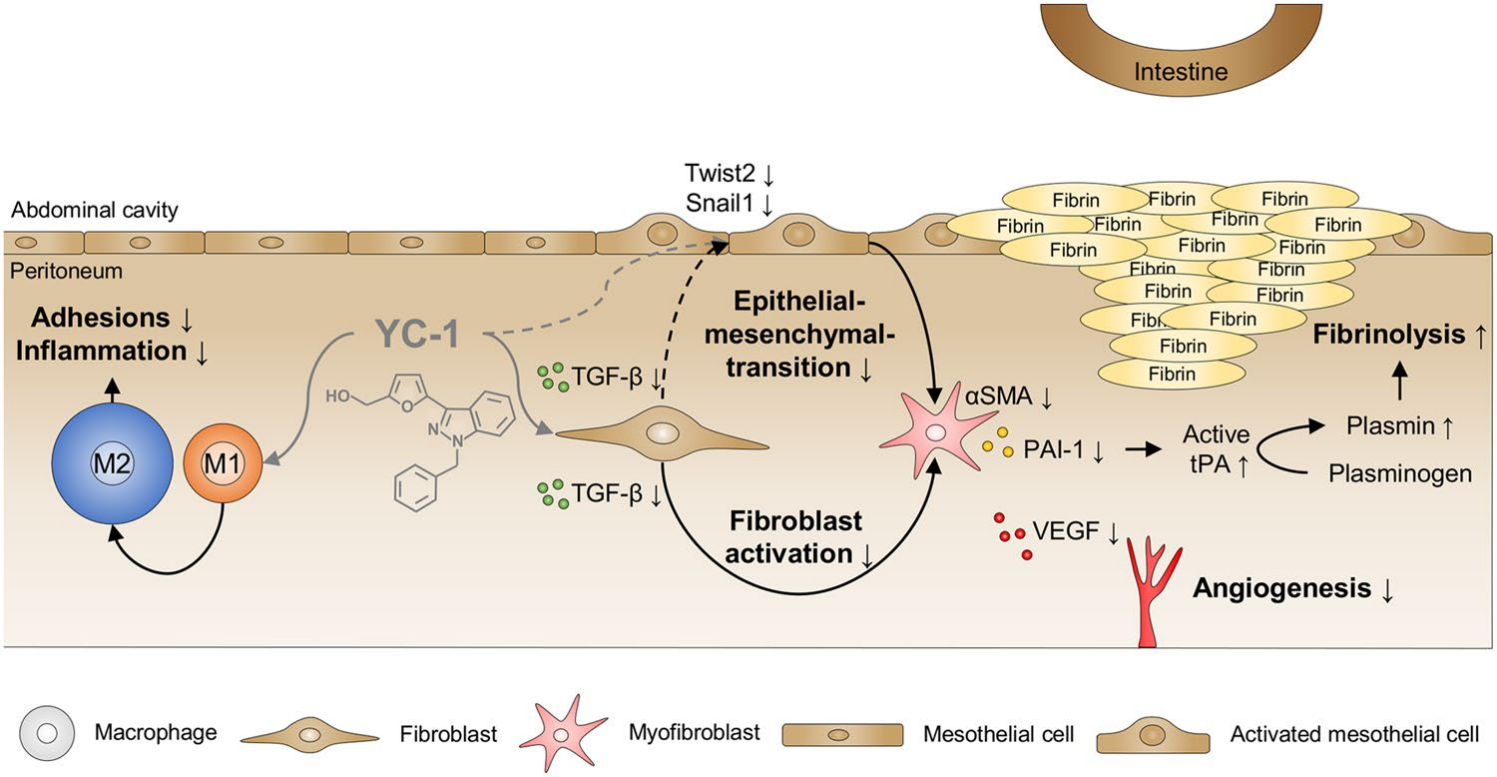
Putative mechanisms mediating anti-adhesive effects of YC-1. Intraperitoneal YC-1 counteracts adhesion-triggering inflammation by diverting pro-inflammatory M1-differentiation of macrophages towards immuno-modulatory M2-differentiation (*left*). In addition, YC-1 attenuates EMT-dependent recruitment and activation of peritoneal fibroblasts (*middle*). Reduced formation of activated, pro-fibrotic myofibroblasts may result in attenuated secretion of plasminogen activator inhibitor (PAI-1) and vascular endothelial growth factor (VEGF), thus enhancing fibrinolysis via active tissue plasminogen activator (tPA), and mitigating angiogenesis, respectively (*right*). These effects cooperatively counteract postoperative adhesion formation.

Here, we demonstrate that intraperitoneal treatment with a small molecule HIF-inhibitor, YC-1, reduces the formation of postoperative peritoneal adhesions in a preclinical rodent model. Further expression studies and *in vitro* data suggest that, on the cellular level, this effect is cooperatively mediated by various HIF-1α-dependent mechanisms (Fig. 6), comprising attenuated pro-inflammatory differentiation of macrophages, reduced recruitment and activation of peritoneal fibroblasts, mitigated EMT, and putatively also enhanced fibrinolysis and impaired angiogenesis.

An important mechanism appears to reside in the drug’s potential to counteract pro-inflammatory (M1-) polarization of peritoneal macrophages, while favouring immuno-modulatory (M2-) polarization. Current research has acknowledged that the peritoneal inflammatory response determines the extent of postoperative adhesions^3,27^. Macrophages, in this context, represent a heterogeneous cell population, since their adhesion-promoting properties depend on their polarization state^28^. It has been shown that M2-polarization of macrophages protects against peritoneal adhesions^4^. Furthermore, inhibition of Th1-cells, which is linked to consecutively attenuated M1-polarization of macrophages, reportedly ameliorates, whereas stimulation of Th1-cells augments adhesion formation, collectively supporting the notion that attenuated M1-polarization of macrophages is partly responsible for the observed anti-adhesion effects of YC-1^29^. We do not intend to rule out that alternative effects (such as YC-1’s property to suppress the activity of NF-κB^30^) likewise contribute, however, our results indicate that YC-1 dampens the peritoneal inflammatory response by rather selectively targeting HIF-1α in macrophages, while leaving HIF-2α-levels widely unaffected. This is consistent with previous reports that HIF-1α augments pro-inflammatory M1-polarization, while HIF-2α promotes an immune-evasive M2-phenotype^14,31–33^. The significance of HIF-1α in boosting pro-inflammatory macrophage activation has been highlighted by various studies applying gene-deficient animal models^31–33^. Our present study demonstrates that this effect can be exploited by pharmacological interference with HIF-1α in a therapeutically relevant setting.

Impaired recruitment and activation of fibroblasts likewise seemed to contribute to reduced adhesion formation in YC-1-treated mice. Inflammation- and hypoxia-triggered recruitment of fibroblasts from various sources is crucial for the formation of fibrous adhesions^2,34^. Mesothelial cells, which trans-differentiate to fibroblasts in a process referred to as epithelial-mesenchymal-transition (EMT; or mesothelial-mesenchymal-transition, MMT), contribute one such source^15,16^. We detected suppressed expression of EMT-inducing genes such as Twist2 in peritoneal adhesion tissue, and reduced abundance EMT-inducing proteins in peritoneal fluid of YC-1 treated mice, collectively suggesting that YC-1-treatment attenuates EMT of peritoneal cells. Yet, hypoxia not only triggers EMT, but also promotes an adhesion phenotype of peritoneal (myo)fibroblasts, which is characterized by upregulation of genes such as TGF-β and αSMA and PAI-1^13,35–38^. This molecular signature of activated myofibroblasts is substantially promoted by HIF-1α and, as indicated by our *in vitro* studies, effectively reverted by its inhibitor YC-1. Interestingly, we could selectively reproduce these effects of YC-1 in fibroblasts by interfering with the expression of HIF-1α, but not HIF-2α, suggesting that they are HIF-1α-dependent. It is in this context worthwhile to mention that the effects of hypoxia on the activation of resident fibroblasts seem to be tissue-specific. While hypoxia-exposure activates hepatic- and pulmonary fibroblasts, opposite effects have been observed in dermal or cancer-associated fibroblasts, indicating that the anti-fibrotic properties of YC-1 in the peritoneum cannot necessarily be extrapolated to comparable effects in other fibrotic disorders^39–42^. At least in the liver and kidney, however, anti-fibrotic properties have been assigned to YC-1 and its derivatives^12,43^.

Beyond its cell-specific effects on macrophages and fibroblasts, YC-1 seems to augment peritoneal fibrinolysis. Postsurgical reduction in peritoneal fibrinolytic activity, causing an imbalance between fibrin formation and -dissolution, promotes adhesions^3,44^. By enhancing tissue plasminogen activator (tPA), YC-1 seems to tip this balance in favour of fibrinolysis (Fig. 6). tPA is the principal activator of plasminogen to form plasmin, which degrades peritoneal fibrin deposits. Albeit we did not measure fibrinolytic activity directly, we observed that YC-1 repressed the peritoneal release of plasminogen activator inhibitor (PAI-1), a HIF-1α target that represents the most important inhibitor of tPA^3^. Finally, our findings indicate that treatment with the HIF-inhibitor YC-1 antagonizes angiogenesis, which is necessary to transform fibrin deposits into permanent fibrous adhesions^26^. This finding does not appear surprising, given HIF-1α’s stimulatory effects on VEGF, the master regulator of de-novo blood vessel formation, and on other pro-angiogenic molecules^9^.

While our findings suggest that YC-1 potentially alleviates peritoneal adhesion formation via various distinct and cell-specific mechanisms, it is on the basis of our present data not possible to discern which of those is paramount in mediating YC-1’s anti-adhesive properties. It is, however, conceivable that the particular strength of the drug resides in its potential to simultaneously target the inflammatory, fibrogenic, fibrinolytic and angiogenic systems by acting on various cell types that determine the extent of adhesion formation, such as macrophages and peritoneal fibroblasts. This unique property could make drugs antagonizing HIF-1α (of which YC-1 is a prototype) outstanding therapeutic tools for the prevention of peritoneal adhesions.

As discussed above, most of YC-1’s anti-adhesion effects may be attributed to its HIF-1α-inhibiting properties. Yet, YC-1 does not exclusively target HIFs. It was originally characterized as a stimulator of soluble guanylate-cyclase (sGC) with anti-thrombotic effects, and served as a lead structure in the development of sGC activating drugs to treat pulmonary hypertension^45,46^. Remarkably, in all *in vitro* assays applied throughout this study, YC-1 was administered at dosages well below those required to stimulate sGC, favouring the notion that the drug’s property to reduce peritoneal adhesions relies predominantly on its activity towards HIF^47^. Importantly, in this context, animals exposed to intraperitoneal YC-1-treatment did not develop any overt, systemic side effects that could be attributed to systemic activation of sGC, or to systemic inhibition of the HIFs.

Regardless the limitations of a preclinical animal model, this study provides first evidence that intraoperative treatment with HIF-inhibiting compounds such as YC-1 efficiently reduces postoperative adhesion formation. Of clinical relevance, YC-1 attenuated adhesions already upon single intraperitoneal application, indicating that topical, peritoneal lavage during surgery represents a suitable mode of administration, which is likewise feasible in laparoscopic procedures. On the one hand, it has been repeatedly demonstrated that single peri-operative administration of certain agents or drugs is sufficient to unfold anti-adhesion effects in pre-clinical settings^24,48^. On the other hand, this raises important questions concerning optimal dosing and application routes for YC-1-treatment in the context of adhesion prophylaxis. While the purpose of our present study was to demonstrate the anti-adhesion effects of the HIF-inhibitor YC-1, answering these crucial questions will mandate further in-depth dose-response studies, as well as analyses of the drug’s pharmacokinetics and biodistribution.

## Methods

### Animal models

All experiments were approved by the local animal welfare committee (Regierungspräsidium Karlsruhe) under application number G-83/15 and carried out in accordance with the criteria outlined in the “Guide for the Care and Use of Laboratory Animals” by the National Academy of Sciences. Up to four animals were kept in one cage under specific pathogen-free conditions with food and water ad libitum and a 12-hour light/dark cycle was obtained. All animals were randomly assigned to different treatment groups and operated by the same surgeon. *In vivo* experiments were carried out at independent time points.

Female Balb/c-mice (9–16 weeks) underwent daily intraperitoneal (i.p.) injections of YC-1 (20 mg/kgBW; Cayman Chemicals, Hamburg, Germany; 25 mg YC-1 dissolved in 5 ml DMSO/water) or vehicle (DMSO, Sigma-Aldrich, Taufkirchen, Germany). Animals were randomly assigned to either group. Three days after the beginning of treatment, mice were anaesthetized with isoflurane (Isothesia, Henry Schein, Hamburg, Germany) and peritoneal adhesions were induced applying the ischemic buttons model creating four buttons per animal with 4-0 Prolene (Ethicon, Norderstedt, Germany) as ligature material^49^. The peritoneum and skin were closed with 6-0 PDS II (Ethicon). All animals received a single subcutaneous injection of buprenorphine (0.05 mg/kgBW) to control postoperative pain. Daily YC-1 injections were continued for seven days (“pretreatment” scheme; Fig. 1a, *left*). All animals were sacrificed by cervical dislocation on postoperative day seven. An established score was used for blinded grading of postoperative adhesions (Supplementary Table S2)^11^. Histological and RT-qPCR-based analysis was conducted applying tissue isolated from these mice.

Alternatively, mice received one single intraoperative lavage with YC-1 (20 mg/kgBW; prepared as indicated above) or vehicle during adhesion induction (“lavage” scheme; Fig. 1a, *middle*). In detail, animals received a single dosage of YC-1 before closing the abdomen.

For assessment of wound healing, mice were treated with YC-1 (20 mg/kgBW) for three days prior to laparotomy. YC-1-treatment was continued for seven days following laparotomy, and mice were subsequently sacrificed for assessment of abdominal wounds (Supplementary Fig. 1c). Laparotomy scars were cut into transverse stripes of 3 mm width using a microtome blade. Two resultant wound stripes per animal were subsequently fixed and pulled apart in a tensiometer. The average maximum wound breaking strength was measured as the greatest weight load until rupture occurred^50^. Collagen deposition was histologically quantified by measuring the shortest distance between the epidermal-dermal and dermal-fat junctions in the subcutis of Masson-Trichrome-Goldner stained laparotomy scar sections^51^.

To assess short-term effects of YC-1, animals were randomized to undergo adhesion induction concomitantly with either single intraoperative YC-1- or vehicle-lavage. Twenty-four hours afterwards, pimonidazole (60 mg/kgBW; Hypoxyprobe, Burlington, USA) was administered and peritoneal lavage fluid was collected (“short term” scheme, Fig. 1a, *right*). Subsequently, inflammatory cytokines, PAI-1 and active tPA were determined in these animals.

To study side effects, animals were sham-operated and i.p. injections of 20 mg/kgBW YC-1 or vehicle were administered in a randomized fashion. After 24 hours, mice were sacrificed and peritoneum, liver and heart were harvested. Plasma samples were collected by punction of the caudal vena cava. Blood cell counts, transaminases and creatinine levels were determined by the central laboratory of the University Hospital Heidelberg. Erythropoietin- (EPO-) levels in heparin plasma were determined by ELISA (R&D Systems, Wiesbaden, Germany).

### Histology and immunohistochemistry

Paraffin sections (as illustrated in Supplementary Fig. 5a) were subjected to Masson-Trichrome-Goldner staining (Merck Millipore, Darmstadt, Germany) to label collagen fibres. For immunohistochemistry, antigens were retrieved with target retrieval solution (Dako, Hamburg, Germany) and blocked with serum derived from the same animal as the secondary antibody (Vector laboratories, Burlingame, USA). The following primary antibodies were used: CD45 (1:100; 553076, BD Pharmingen, Heidelberg, Germany), F4/80 (1:200; MCAP497, AbD Serotec, Puchheim, Germany), αSMA (1:200; ab5694, Abcam, Cambridge, UK) and CD31 (1:100; ab28364, Abcam). Appropriate secondary antibodies (BA-1100, BA-4001; Vector Laboratories) were added and amplified with TSA^TM^ Indirect (Perkin Elmer, Rodgau, Germany) prior to DAB-labelling (Dako). Positively stained cells or areas were quantified by two independent, blinded investigators applying 20 standardized high-power-fields (Supplementary Fig. 5b) using an Axiostar Plus Microscope (Carl Zeiss, Jena, Germany) and ImageJ software (National Institutes of Health, Bethesda, USA).

### Quantitative real-time PCR

Total RNA was isolated using RNeasy Mini Kit (Qiagen, Hilden, Germany) and transcribed into cDNA applying the Improm-II-Reverse Transcription System (Promega, Mannheim, Germany). qRT-PCR was performed using Light Cycler ® 480 SY Green Master (Roche, Mannheim, Germany). Specific primers were obtained from Thermo Fisher Scientific (Darmstadt, Germany; Supplementary Table S3). Transcript levels were calculated relative to the house keeping gene β-Actin.

### Analysis of peritoneal lavage and tissue lysates

Peritoneal fluid was analysed with a Mouse XL Cytokine Array (R&D Systems) according to the manufacturer’s instructions. Chemiluminescence was detected with Fusion SL2-3500.WL (Vilber Lourmat, Suebia, Germany). Densiometric analysis of dots representing each cytokine was performed with ImageJ. Background signal was subtracted, and expression of each cytokine was normalized to its own positive control. Levels of IL-6 (R&D Systems), PAI-1 (Thermo Fisher Scientific), active tPA (LifeSpan BioScience, Seattle, USA) and were quantified by ELISA. Absorbance was measured at 450 and 540 nm using a 96-well plate reader (Tecan, Crailsheim, Germany).

### Cell culture experiments

J774.A1- and NIH-3T3 cells were purchased from ATCC (LGC Standards, Wesel, Germany) and cultured in DMEM (Sigma-Aldrich) with 10% FCS and 1% Penicillin/Streptomycin. Unless otherwise indicated, cells were cultured at 5% CO_2_ and ambient oxygen concentrations at 37 °C. Peritoneal fibroblasts were isolated as described elsewhere^52^. Briefly, peritoneal tissue was minced and digested with collagenase (Sigma-Aldrich). Primary fibroblasts were cultured in EMEM (Sigma-Aldrich) with 15% FCS, 1% Penicillin/Streptomycin and 2 mM L-Glutamine (Sigma-Aldrich). To determine cell identity, cells were grown on coverslips, fixed and labelled with a primary antibody labelling fibroblast-specific protein 1 (FSP-1, 1:100; ab27957, Abcam), and a TRITC-labelled secondary antibody (1:100; T6778, Sigma-Aldrich)^53^.

Fibroblasts were treated with different concentrations of YC-1 (0.1 μM, 1 μM and 10 μM) or 1% DMSO while incubated in a hypoxia chamber (Billups-Rothenberg, San Diego, USA) at 0.75% oxygen for 16 h (for mRNA analysis and nuclear extracts) or 24 h (for protein analysis). Addition of 1% DMSO did not impair cell viability. For immunocytochemistry, cells were grown on coverslips, fixed, stained for αSMA (1:100; ab5694, Abcam) and labelled with a TRITC-conjugated secondary antibody (1:100, Sigma-Aldrich). For determination of PAI-1 levels in cell culture supernatant, fibroblasts were incubated with starvation medium (5% FCS) for 24 h while treated with DMSO or 10 μM YC-1 under normoxia and hypoxia. Subsequently, supernatant was collected and analysed by ELISA (Thermo Fisher Scientific).

J774.A1 cells were differentiated into macrophages with 200 nM phorbol myristate acetate (PMA; Sigma-Aldrich) for 48 h and subsequently treated with 100 ng/ml lipopolysaccharide (LPS; Sigma-Aldrich) and 10 μM YC-1 or 1% DMSO under normoxia or hypoxia (0.75% oxygen) for 16 h. To measure phagocytosis, cells were incubated with pHrodo® Red E. coli BioParticles® Conjugate (Thermo Fisher Scientific), counter-stained with AlexaFluor488-phalloidin (Thermo Fisher Scientific), and fluorescence intensity was assessed at 535/595 nm.

Bone marrow-derived macrophages (BMDMs) were isolated as previously reported^14^. Briefly, femur and tibia were flushed with PBS and cells were differentiated in RPMI-1640 (Sigma-Aldrich) with 10% FCS, 1% Penicillin/Streptomycin, 2 mM L-Glutamine and 10 ng/ml mM-CSF (R&D Systems) for seven days prior to experiments. Immunostaining for F4/80 (1:100; ab6640, Abcam) was applied to confirm macrophage identity^54^. Analogous to J774.A1 cells, BMDMs were treated with 10 μM YC-1 or DMSO with or without 100 ng/ml LPS for 16 h und normoxia and hypoxia (0.75% O_2_) prior to RNA isolation or phagocytosis assay.

### Transient HIF-knockdown

NIH-3T3 fibroblasts were transfected with four siRNAs (Flexi Tube Gene solution, Qiagen) targeting murine *Hif1a* (catalogue number GS15251; SI00193032, SI00193025, SI00193018, SI00193011) or *Epas1* (catalogue number GS13819; SI04894148, SI04892867, SI02736636, SI02712192) using Lipofectamine RNAiMAX (Thermo Fisher Scientific) for 24 h. Cells were subsequently exposed to 0.75% oxygen for 16 h. Knockdown efficiency was determined via RT-qPCR and Western blotting. Cells transfected with AllStars Negative Control siRNA (Qiagen; SI03650318) served as control.

### Western Blotting

Total protein was isolated using T-PER™ Tissue Protein Extraction Reagent (Thermo Fisher Scientific) for tissue and RIPA lysis buffer (Merck Millipore) for whole-cell lysates. Nuclear extracts were prepared using NE-PER reagents (Thermo Fisher Scientific). Primary antibodies detecting TGF-β (1:1,000; ab66043, Abcam), Vinculin (1:2,000; ab73512, Abcam), HIF-1α (1:1,000; NB100-479, Novus Biologicals, Wiesbaden, Germany), HIF-2α (1:500; NB100-122, Novus Biologicals) and Histone H3 (1:1,000; 9715, Cell Signaling Technology, Frankfurt, Germany) as well as HRP-conjugated secondary antibodies (1:5,000; sc-2004 and sc-2005, Santa Cruz, Heidelberg, Germany) were applied. Chemiluminescence was detected with Fusion SL2-3500.WL and semi-quantitative densiometric analysis was carried out with ImageJ.

### Statistics

All values are shown as arithmetic mean ± standard error of the mean. Figure panels reflect data from one of at least two representative experiments. Normal distribution of data was confirmed applying the Shapiro-Wilk test. Group differences were evaluated by two-tailed Student’s *t*-test, ANOVA with appropriate post-hoc test or Fisher’s exact test where applicable using GraphPad Prism 5.00 (GraphPad Software, San Diego, USA). *P* values < 0.05 were considered significant. The datasets generated during and/or analysed during the current study are available from the corresponding author on reasonable request.

## Electronic supplementary material

Supplementary information
