# Supplementary material for: Pharmacological HIF-inhibition attenuates postoperative adhesion formation
Source: Sci Rep. 2017 Oct 13;7:13151. doi: 10.1038/s41598-017-13638-z (PMC5640636; doi:10.1038/s41598-017-13638-z)

PHARMACOLOGICAL HIF-INHIBITION ATTENUATES POSTOPERATIVE ADHESION FORMATION

Moritz J. Strowitzki, MD, Alina S. Ritter, Praveen Radhakrishnan, PhD, Jonathan M. Harnoss, MD, Vanessa M. Opitz, Marvin Biller, Julian Wehrmann, Ulrich Keppler, MD, Jana Scheer, Markus Wallwiener, MD, Thomas Schmidt, MD, PhD, Alexis Ulrich, MD, and Martin Schneider, MD

SUPPLEMENTARY NOTES

Supplementary Note: For technical reasons, siRNA-mediated knockdown of the HIFs was not feasible in primary fibroblast cultures, which became apoptotic upon exposure to transfection reagents (as indicated by cell rounding and excessive detachment). We therefore applied a standard, immortalized murine embryonic fibroblast line (NIH-3T3) as a surrogate experimental model. qRT-PCR and Western blotting revealed that siRNA-mediated silencing efficiently suppressed the expression of HIF-1α and HIF-2α in NIH-3T3 cells (Supplementary Fig. 4*f* and *g*). Similar to peritoneal fibroblasts, YC-1 treatment likewise attenuated the expression of TGF-β and PAI-1 in NIH-3T3 fibroblasts (Supplementary Fig. 4*h* and *j*).

SUPPLEMENTARY FIGURE LEGENDS

Supplementary Fig. 1. *Side effects of YC-1-treatment.* ***(a, b)*** Daily body weight measurements of mice undergoing adhesion induction (arrow) upon either pretreatment (*a*) or single lavage (*b*) with YC-1 or DMSO (Ctrl). Neither regime of YC-1-treatment induced significant weight loss ($n = 9$). ***(c)*** Experimental timeline to assess effects of YC-1 on wound healing. Mice were pretreated for 3 days prior to sham laparotomy with YC-1 (20 mg/kgBW) or vehicle (DMSO/water). Treatment was continued until evaluation of wounds on POD7. ***(d)*** Determination of mean laparotomy wound breaking strength in mice treated with vehicle (Ctrl) or YC-1 (ns, non significant; $n = 8$). **(*e*)** Representative Masson-Trichrom-Goldner stainings (*left*) and histomorphometric quantification (*right*) of collagen deposits within laparotomy scars of mice treated with YC-1 or vehicle (Ctrl). (ns, non significant; $n = 8$). Scale bars = 200 μm. ***(f)*** Experimental schedule for studying side effects of YC-1-treatment. Mice were sham operated and intraoperatively treated with YC-1 (20 mg/kgBW) or vehicle (DMSO/water). Twenty-four hours later, animals were sacrificed for blood- and tissue sampling. ***(g, h)*** qRT-PCR analyses, revealing mRNA expression levels of selected HIF-target genes (PDK-1, VEGF, Glut1, CyclinD1) in livers (*g*) and hearts (*h*) of mice treated with YC-1 or vehicle (Ctrl) ($n = 5$).

Supplementary Fig. 2. *Effects of YC-1 on macrophages.* ***(a, b)*** Representative imaging (*a*) and quantification (*b*) of fluorescent bacterial particles (pHrodo, yellow), phagocytosed by J774.A1 macrophages upon treatment with YC-1 or vehicle (DMSO) in hypoxic culture conditions. Phalloidin-immunocytochemistry (green) was used for cytoskeletal counterstaining ($P \leq 0.05$, $n = 8$). Scale bars = 50 μm. ***(c)*** Representative immunocytochemistry for the macrophage marker F4/80 (red) and DAPI (cell nuclei,

blue), confirming the macrophage identity of BMDMs isolated from mice after seven days of differentiation with M-CSF. Scale bars = 100 μm. ***(d, e)*** Representative imaging (*d*) and quantification (*e*) of fluorescent bacterial particles (pHrodo, yellow), phagocytosed by BMDMs upon treatment with YC-1 or vehicle (DMSO) in hypoxic culture conditions. Phalloidin-immunocytochemistry (green) was used for cytoskeletal counterstaining (** $P \leq 0.01$, * $P \leq 0.05$, $n = 6$). Scale bars = 50 μm.

Supplementary Fig. 3. *YC-1 affects the expression of EMT-related proteins in peritoneal fluid.* ***(a, b)*** Antibody array, revealing the abundance of the EMT-inducing cytokines, EGF, FGF-1, HGF (*a*), and the repressors of IGF, IGFBP1-6 (*b*), in peritoneal fluid of mice treated with YC-1 or vehicle (Ctrl), one day after adhesion induction (*** $P \leq 0.001$, $n = 4$; ANOVA with Bonferroni post-hoc tests).

Supplementary Fig. 4. *YC-1 attenuates fibroblast activation.* ***(a)*** Representative immunocytochemistry for fibroblast-specific protein 1 (FSP-1, red) and DAPI (cell nuclei, blue), confirming the fibroblasts-phenotype of primary peritoneal fibroblasts isolated from mice. Scale bars = 100 μm. ***(b, c)*** Western blots of nuclear extracts, revealing protein levels of HIF-1α (*b*) and HIF-2α (*c*) in primary fibroblasts treated with vehicle (DMSO) or YC-1 under normoxic (*left*) or hypoxic (*right*) culture conditions. Note reversal of hypoxic HIF-1α -, but not HIF-2α stabilization upon YC-1-treatment. Histone H3 was used as loading control. ***(d, e)*** Western blots of nuclear extracts, revealing protein levels of HIF-1α (*d*) and HIF-2α (*e*) in embryonic NIH-3T3 fibroblasts treated with vehicle (DMSO) or YC-1 under normoxic (*left*) or hypoxic (*right*) culture conditions. Histone H3 was used as loading control. ***(f)*** qRT-PCR, confirming efficient repression of HIF-1α - (*left bars*) and HIF-2α (*right bars*) mRNA transcript levels in

NIH-3T3 fibroblasts following siRNA-mediated knockdown (*** $P \leq 0.001$, $n = 12$). Ctrl, control-transfected cells. ***(g)*** Western blot, confirming successful repression of HIF-1α protein levels in hypoxia-treated NIH-3T3 fibroblasts following siRNA-mediated knockdown of HIF-1α. β-Actin was used as a loading control. ***(h)*** qRT-PCR to assess TGF- β mRNA expression in NIH-3T3 fibroblasts treated with vehicle (DMSO) or YC-1 (1 μM) at hypoxic culture conditions. TGF- β expression is suppressed upon YC-1-treatment (* $P \leq 0.05$, $n = 6$). ***(i)*** ELISA measurement, revealing PAI-1 protein concentrations in cell culture supernatant from primary fibroblasts treated with vehicle (DMSO) or YC-1 (10 μM) under normoxia and hypoxia (*$P \leq 0.05$, $n = 6$). ***(j)*** qRT-PCR, revealing suppressed PAI-1 mRNA expression in hypoxic NIH-3T3 fibroblasts upon treatment with 1 μM YC-1 (* $P \leq 0.05$, $n = 6$).

Supplementary Fig. 5. *Microscopic appearance of ischemic peritoneal buttons.* ***(a)*** Scheme of ischemic peritoneal button. Dotted black lines indicate the orientation of serial histological sections. ***(b)*** Representative hematoxylin and eosin staining of an ischemic peritoneal button, depicting the necrotic core (#), the former position of the suture (*) and the fibrous adhesion area (+) with adjacent fatty tissue (§). The black arrow marks the region of interest, which was further analyzed by immunohistochemistry and histomorphometric quantification (as depicted in Figures 2*a, b*, 4*c* and 5*e*). Scale bar = 500 μm.

SUPPLEMENTARY TABLES

Supplementary Table S1. Blood values of sham-operated mice on postoperative day 1.

Blood value		Ctrl		YC-1		P-value
		**Mean**	**SEM**	**Mean**	**SEM**	
Erythrocytes	[/pl]	6.50	± 0.85	6.68	± 0.49	**0.89**
Hemoglobin	[g/dl]	11.96	± 1.52	12.48	± 0.72	**0.81**
Hematocrit	[l/l]	0.34	± 0.05	0.35	± 0.02	**0.96**
Erythropoietin	[pg/ml]	134.35	± 7.43	166.12	± 23.93	**0.31**
Leucocytes	[/pl]	3.40	± 0.62	2.48	± 0.29	**0.31**
Thrombocytes	[/pl]	1035.00	± 48.54	1082.75	± 38.44	**0.53**
Creatinine	[mg/dl]	0.16	± 0.03	0.15	± 0.02	**0.85**
GOT	[U/l]	63.75	± 6.92	51.80	± 2.63	**0.24**
GPT	[U/l]	40.50	± 7.63	28.00	± 7.04	**0.33**

Ctrl control group; *GOT* glutamic oxaloacetic transaminase; *GPT* glutamic pyruvic transaminase; *POD* postoperative day; *SEM* standard error of the mean. *n = 5*. Student's t-test.

Supplementary Table S2. Qualitative and quantitative score for grading of postoperative adhesion formation.

Extent		Type		Tenacity		Total
0	no adhesions	0	no adhesions	0	no adhesions	**= Extent + Type + Tenacity**
1	1%–25%	1	filmy	1	easily fall apart	
2	26%–50%	2	dense	2	require traction	
3	51%–75%	3	capillaries present	3	require sharp dissection	
4	76%–100%					

Supplementary Table S3. Primer sequences.

Gene	Protein	Forward Sequence (5’ to 3’)	Reverse Sequence (5’ to 3’)
Acta2	αSma	CTTTTCCATGTCGTCCCAGT	AATGGCTCTGGGCTCTGTAA
Actb	β-Actin	GGTCATCACTATTGGCAACG	GGCATAGAGGTCTTTACGGATG
Ccnd1	Cyclin D1	TGGAAAGAAAGTGCGTTGTG	CGGATGAGAACAAGCAGACC
Epas1	Hif-2α	CAGCTTCCTTCGGACACATAAG	CTGGTCGGCCTCAGCTTC
Hif1a	Hif-1	GGGCTTTCAGATAAAAACAGTCCAT	TGAGCTCACATCTTGATAAAGCTTCT
Il6	Il-6	ACAAAGCCAGAGTCCTTCAGAG	TCCTTAGCCACTCCTTCTGTG
Nos2	iNos	GGAAGAAATGCAGGAGATGG	AGCTGCTTTTGCAGGATGTC
Pgk-1	Pgk-1	GCCTTGATCCTTTGGTTGTTTG	GGAAGCGGGTCGTGATGA
Serpine1	Pai-1	TAGCACAGGCACTGCAAAAG	GTTGTGCCGAACCACAAAG
Slc2a1	Glut1	ACGATTGATGAGCAGGAAGC	AATGCAGACTTGTGGCCTCT
Snai1	Snail1	TGGAAAGGCCTTCTCTAGGC	TCAGCAAAAGCACGGTTG
Tgfb1	Tgf-β1	TCCAAACAGATGGCAGAGC	TTCCTGTTGGCTGAGTTGTG
Twist2	Twist2	CACGAGCGTCTCAGCTACG	AGGAGCCACAAGGTTGTCC
Vegfa	Vegf-a	TCACCAAAGCCAGCACATAG	ATGCTTTCTCCGCTCTGAAC
Vim	Vimentin	GCTGCGAGAGAAATTGCAG	TCTTCCTGCAAGGATTCCAC

Supplementary Fig. 1

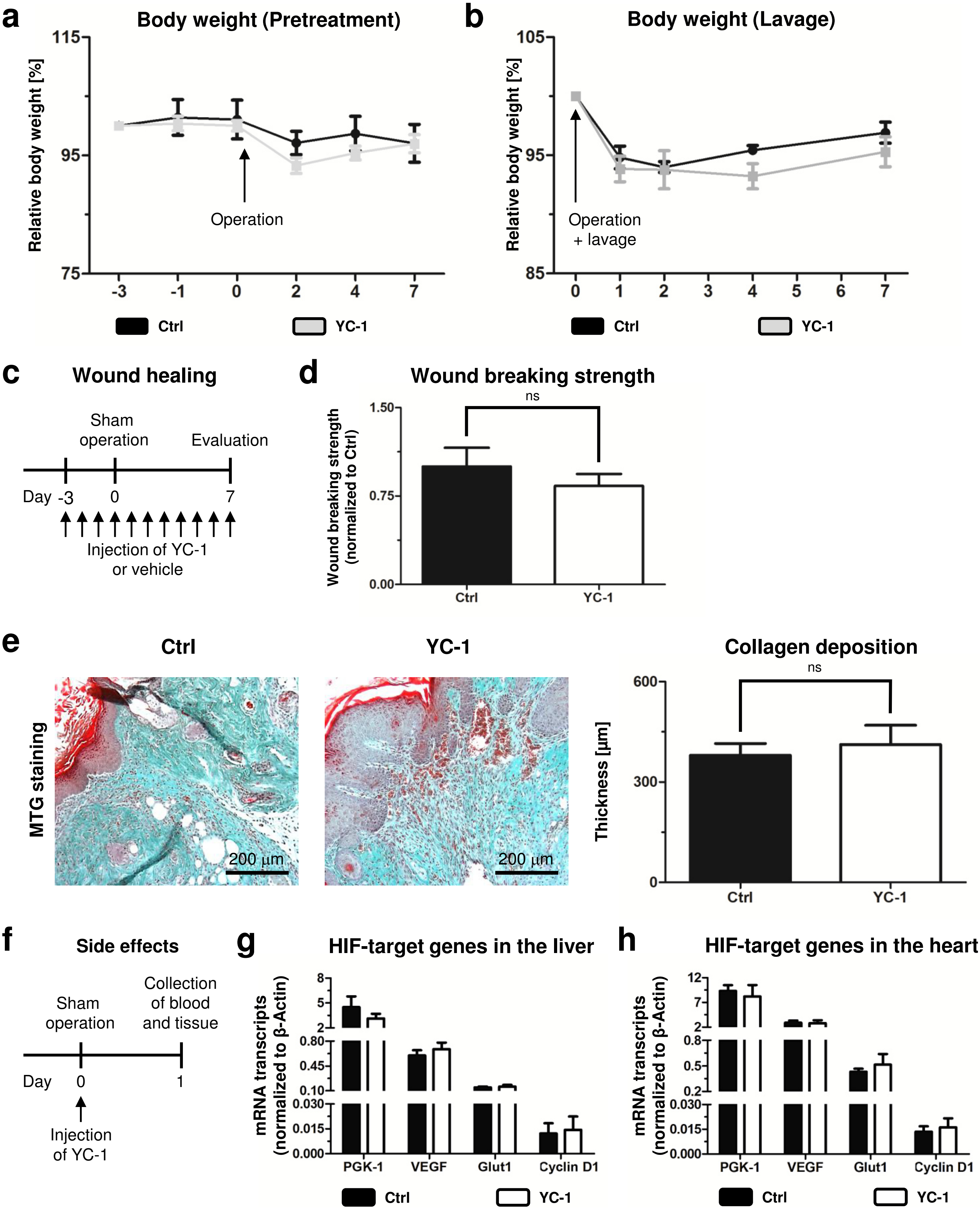

Supplementary Fig. 2

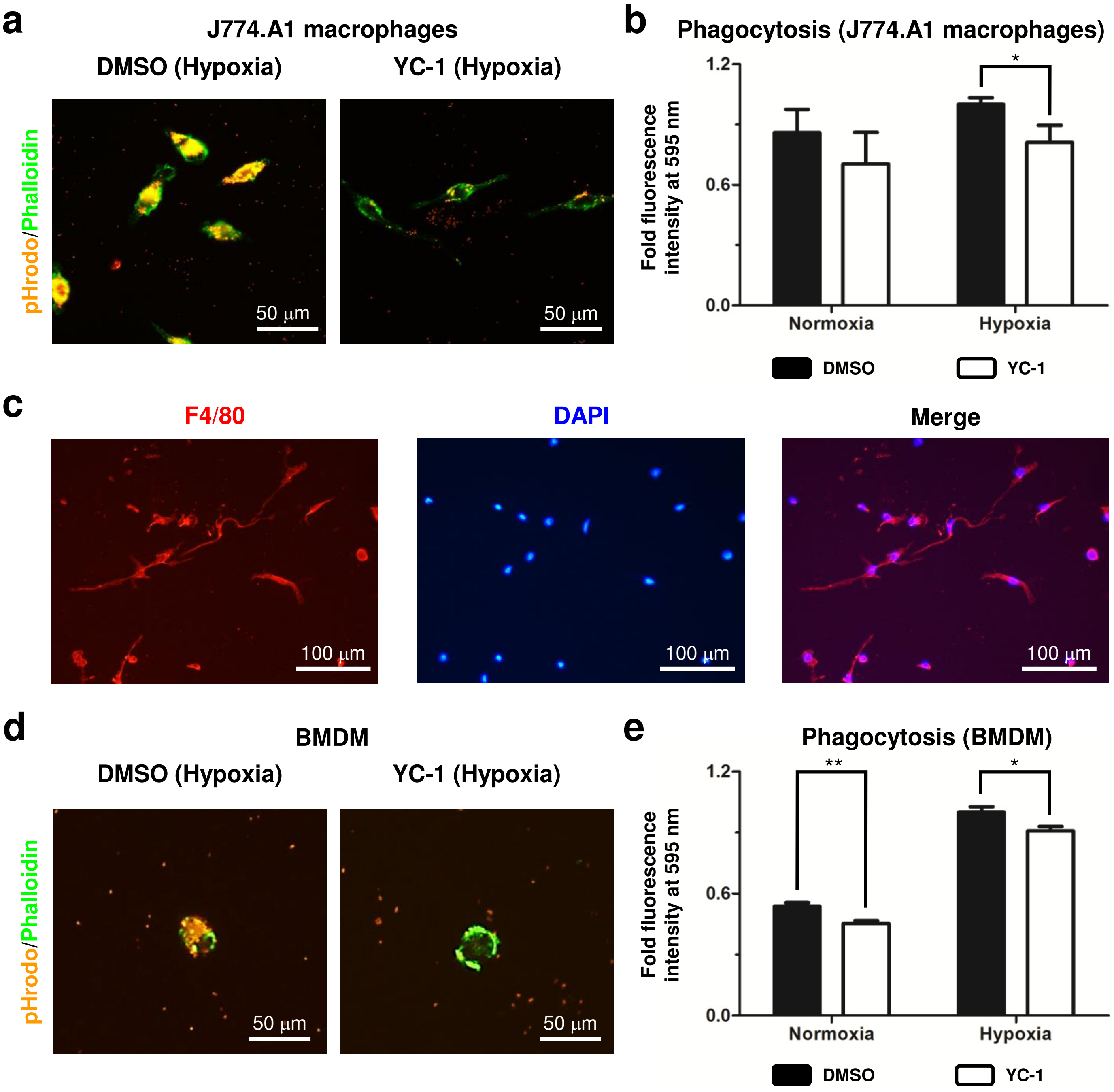

Supplementary Fig. 3

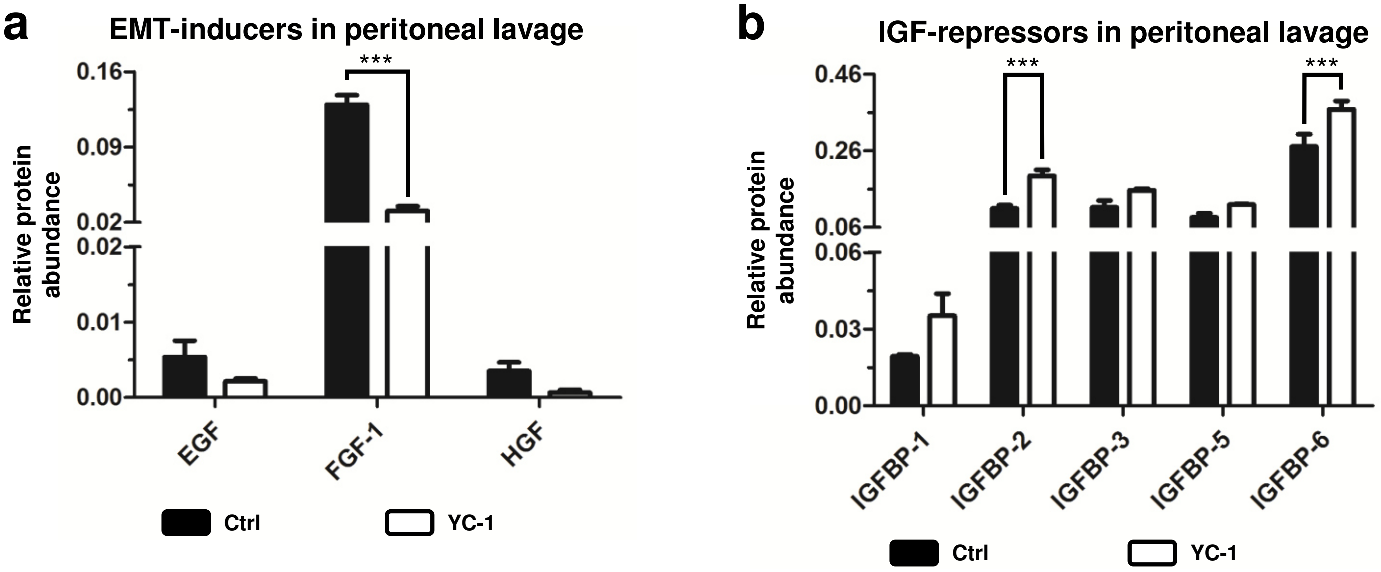

Supplementary Fig. 4

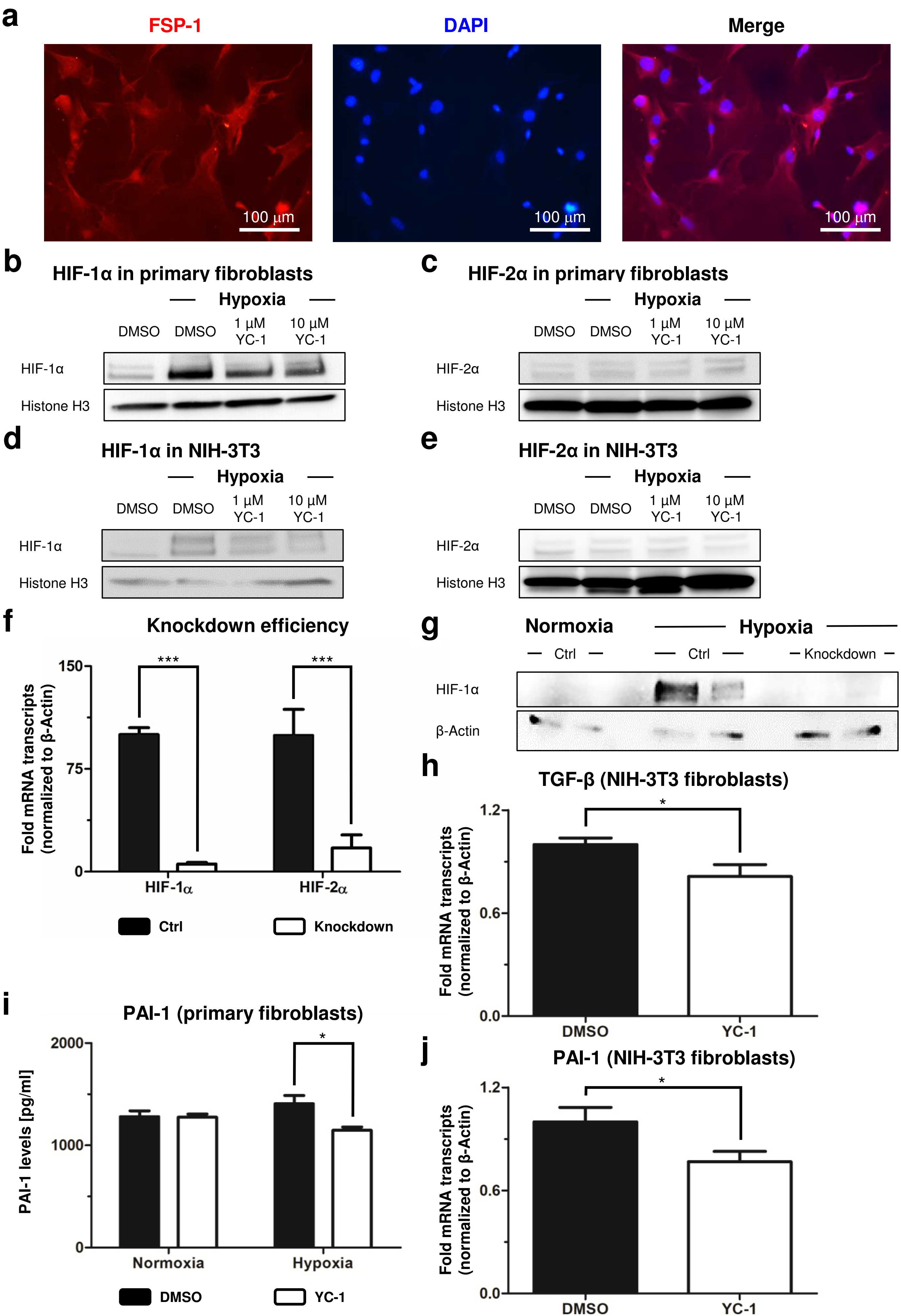

Supplementary Fig. 5

a

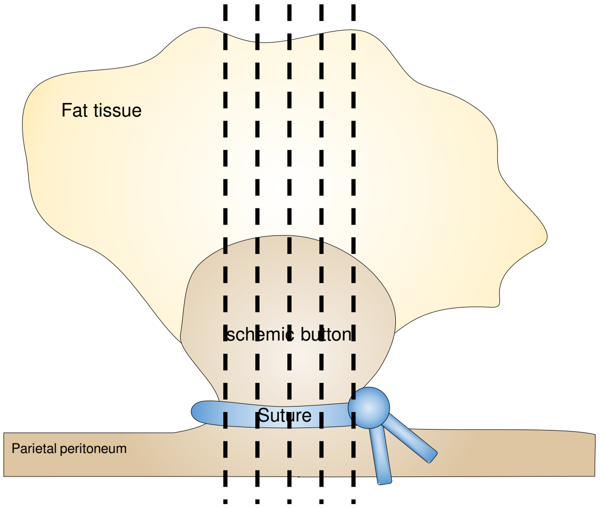

b

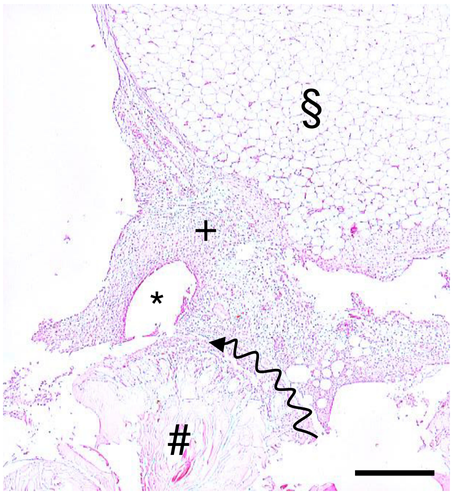

Supplement: Supplementary file 1 — Supplementary information [file 41598_2017_13638_MOESM1_ESM.pdf]
